# Alkali-Soluble Pectin Is the Primary Target of Aluminum Immobilization in Root Border Cells of Pea (*Pisum sativum*)

**DOI:** 10.3389/fpls.2016.01297

**Published:** 2016-09-13

**Authors:** Jin Yang, Mei Qu, Jing Fang, Ren Fang Shen, Ying Ming Feng, Jia You Liu, Jian Feng Bian, Li Shu Wu, Yong Ming He, Min Yu

**Affiliations:** ^1^Department of Horticulture, Foshan UniversityFoshan, China; ^2^College of Resources and Environment, Huazhong Agricultural UniversityWuhan, China; ^3^State Key Laboratory of Soil and Sustainable Agriculture, Institute of Soil Science, Chinese Academy of ScienceNanjing, China; ^4^College of Life Science and Engineering, Foshan UniversityFoshan, China

**Keywords:** pea (*Pisum sativum*), alkali-soluble pectin, chelate-soluble pectin, root border cells (RBCs), aluminum adsorption/desorption

## Abstract

We investigated the hypothesis that a discrepancy of Al binding in cell wall constituents determines Al mobility in root border cells (RBCs) of pea (*Pisum sativum*), which provides protection for RBCs and root apices under Al toxicity. Plants of pea (*P. sativum* L. ‘Zhongwan no. 6’) were subjected to Al treatments under mist culture. The concentration of Al in RBCs was much higher than that in the root apex. The Al content in RBCs surrounding one root apex (10^4^ RBCs) was approximately 24.5% of the total Al in the root apex (0–2.5 mm), indicating a shielding role of RBCs for the root apex under Al toxicity. Cell wall analysis showed that Al accumulated predominantly in alkali-soluble pectin (pectin 2) of RBCs. This could be attributed to a significant increase of uronic acids under Al toxicity, higher capacity of Al adsorption in pectin 2 [5.3-fold higher than that of chelate-soluble pectin (pectin 1)], and lower ratio of Al desorption from pectin 2 (8.5%) compared with pectin 1 (68.5%). These results indicate that pectin 2 is the primary target of Al immobilization in RBCs of pea, which impairs Al access to the intracellular space of RBCs and mobility to root apices, and therefore protects root apices and RBCs from Al toxicity.

## Introduction

Aluminum (Al) toxicity is one of the main factors limiting plant growth and crop production in acid soils, which account for around 30–40% of the world’s arable land and nearly 50% of potentially arable land (von Uexküll and Mutert, 1995). The rapid inhibition of cell elongation and root growth is primarily due to the accumulation of Al in plant cell walls (Čiamporová, 2002; Kochian, 2003; Ma et al., 2004; Panda et al., 2009; Horst et al., 2010). However, it is still debatable whether Al accumulation in cell walls is an indicator of Al tolerance or Al sensitivity (Ishikawa et al., 2001; Horst et al., 2010; Xia et al., 2010; Zhu et al., 2013). Increasing evidence suggests that the root apoplast plays a vital role in Al toxicity/Al sensitivity (Yang et al., 2008, 2011; Yu et al., 2009; Horst et al., 2010; Jian Li et al., 2011; Zhu et al., 2013). Al exerts its toxic effect on the apoplast through interactions with negatively charged binding sites of the cell wall, which are mainly provided by the carboxylic groups of the pectin matrix (Yang et al., 2008, 2011; Horst et al., 2010). Hemicellulose may also be involved in Al binding and accumulation (Yang et al., 2008, 2011; Zhu et al., 2013). Thus, a decrease in pectin and hemicellulose induced by ammonium leads to a decrease in Al accumulation (Wang et al., 2015). Binding of Al to the cell wall increases cell wall rigidity and decreases its elasticity (Ma et al., 2004). However, Al binding to the cell wall actually provides a shield for the cell under Al toxicity, as Al is more toxic to the symplast than to the apoplast (Yamamoto et al., 2002; Panda et al., 2009; Liu et al., 2014). The mechanism by which plant cells, especially RBCs, tackle the dilemma of Al accumulation in the cell wall is therefore intriguing.

Root border cells, living cells surrounding root apices of most plant species (Hawes et al., 2000; Yu et al., 2009), are considered to perform pivotal roles in protecting root apices from Al toxicity (Miyasaka and Hawes, 2001; Tamás et al., 2005; Xing et al., 2008; Yu et al., 2009; Geng et al., 2011; Shu et al., 2011; Cai et al., 2012, 2013). Significantly more Al was found in RBCs than in root apices when RBCs were kept intact under mist culture (Yu et al., 2009). Further analysis indicated that the majority of Al accumulates in cell walls of RBCs (Yu et al., 2006, 2009). Physical removal of RBCs from Al-treated roots has been shown to increase Al accumulation and root growth inhibition in root tips of barley (*Hordeum vulgare* L.; Tamás et al., 2005), pea (*Pisum sativum*; Yu et al., 2009), rice (*Oryza sativa*; Xing et al., 2008; Cai et al., 2012), and soybean (*Glycine max* L.; Shu et al., 2011; Cai et al., 2013). When subjected to Al toxicity, active responses are observed in RBCs, e.g., an increase in uronic acids and 3-deoxy-D-manno-2-octulosonic acid (KDO) of pectin and mucilage (Miyasaka and Hawes, 2001; Tamás et al., 2005; Yu et al., 2006, 2009; Shu et al., 2011; Cai et al., 2012, 2013) and production of H_2_O_2_ (Tamás et al., 2005), indicating they are fighting back to avoid Al injury to themselves in addition to protecting the root apex. Therefore, how RBCs manage to survive while protecting root tips by hyper-accumulation of Al in cell walls is somewhat of an enigma.

It has been hypothesized that the discrepancy in Al binding of cell wall constituents with contrasting chemical and structural properties (Gilbert, 2010; Altartouri and Geitmann, 2015) determines Al mobility in RBCs of pea, which provides protection for RBCs and root apices under Al toxicity. The aim of the current research was to determine the distribution of Al in cell wall constituents, and the action of Al adsorption and/or desorption on cell walls and pectin, to increase our understanding of the dual roles of cell wall pectin that contribute to protection of RBCs and, concomitantly, root apices.

## Materials and Methods

### Plant Materials

Pea seeds (*P. sativum* L. ‘Zhongwan no. 6’) were immersed in 1.5% sodium hypochlorite for 30 min and then rinsed six times with distilled water in order to remove seeds of poor quality, according to the method of Yu et al. (2006). The seeds with good quality were soaked in 0.5 mM CaCl_2_ for 8 h at 24°C in the dark. Soaked seeds were germinated in 0.5 mM CaCl_2_ (pH 4.5) mist produced from a mist culture device for 24 h (Yu et al., 2006). The emerging radicles were then exposed to 0 or 1.0 mM AlCl_3_ (in 0.5 mM CaCl_2_, pH 4.5) mist for another 24 h. Root apices, cut with scissors to a length of approximately 2 cm, were immersed in 0.5 mM CaCl_2_ solution and gently stirred for 5 min. RBC suspensions were pelleted at 300 g for 5 min and rinsed in 0.5 mM CaCl_2_ solution. Root segments (0–2.5 mm, 2.5–5.0 mm, and 5.0–10.0 mm) were cut with a razor blade after the harvest of RBCs. The RBC pellets and root segments were used for further experiments.

### Sequential Extraction of Cell Wall Constituents

Chemical constituents were sequentially extracted from the samples of RBCs and root segments (0–2.5 mm) following the cell wall preparation procedure of Heim et al. (1991) with slight modification. Distilled water, chloroform/methanol solution, 1% SDS solution, 1% α-amylase, 0.25 M imidazole, 50 mM Na_2_CO_3_ (20 mM CDTA), and 4 mM KOH were used to extract water-soluble materials, lipid-soluble materials, protein and phenols, starch polysaccharide, pectin 1, pectin 2, and hemicellulose, sequentially. All pellets were centrifuged at 3396 × *g* for 10 min and washed twice with ultrapure water. Al was extracted from samples of root apices/RBCs, crude cell wall, pectin 1, pectin 2, hemicellulose, and cellulose in 2 M HCl solution for 48 h as described by Yu et al. (2009). Al content was determined using ICP-AES (IRIS-Advantage, Thermo Elemental, Franklin, MA, USA).

### Adsorption of Al in Cell Wall Materials

Adsorption of Al in cell wall materials was carried out in a mini-column following the methods of Zheng et al. (2004) with modification. Crude cell wall (Cell wall), and cell wall following the removal of pectin 1 (Cell wall-pectin 1) and subsequently pectin 2 (Cell wall–pectin 1, 2) was placed in a 2 mL mini-column equipped with a filter at the bottom and balanced in distilled water (pH 4.5) for 24 h. The adsorption solution consisted of 30 μM AlCl_3_ solution (pH 4.5) and was introduced at a speed of 0.2 mL min^-1^ controlled by a peristaltic pump. Fractions were collected at 10 min intervals until Al concentration was constant. Al concentration was immediately determined by a colorimetric method using pyrocatechol violet and imidazole buffer (Ma et al., 1997).

### Adsorption and Desorption of Al in Pectin

A solution of pectin 1 or 2 (50 μg uronic acids at a density of 10 μg mL^-1^), extracted from RBCs in the absence of Al, was pipetted into dialysis bags with a molecular mass cutoff of 3 kDa and equilibrated in 500 mL distilled water (pH 4.5) for 24 h. Adsorption was carried out by immersing the dialysis bag in 500 mL 30 μM AlCl_3_ (pH 4.5) solution for 24 h with gentle stirring every 2 h. The dialysis bag was then transferred to 500 mL CaCl_2_ solution (0.2 mM, pH 4.5) for desorption for another 24 h after a quick rinse (2 s) to remove surface Al. Preliminary experiments showed that the adsorption or desorption equilibrated within 24 h (data not shown). Al content in the equilibrated adsorption or desorption solution was measured colorimetrically using pyrocatechol violet (Ma et al., 1997).

### Quantification of Uronic Acids

Uronic acids content in pectins 1 and 2 of RBCs was assayed according to the method of Blumenkrantz and Asboe-Hansen (1973) using GalUA (Sigma) as a standard to represent residues of pectin with a negative charge that could bind Al.

### Statistical Analysis

Analysis of variance was performed using the ANOVA procedure of the statistical program SAS 8.1, and means were compared using Duncan’s multiple range test among the treatments at *p* < 0.05.

## Results

### Al in RBCs and Root Segments

**FIGURE 1:**
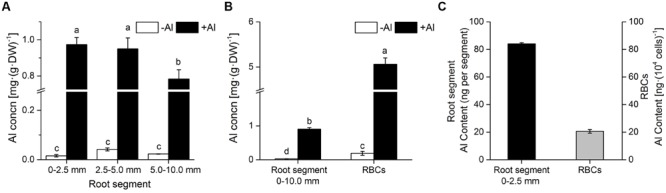
**Accumulation of Al in RBCs and root segments. (A)** Al concentration in root segments (0–2.5 mm, 2.5–5.0 mm, and 5.0–10.0 mm); **(B)** Al concentration in RBCs and root segments (0–10.0 mm); **(C)** Al content in one root apex (0–2.5 mm) and one set of RBCs around the apex. Seedlings were treated with 0 or 1.0 mM AlCl_3_ (in 0.5 mM CaCl_2_, pH 4.0) mist for 24 h. Al in RBCs and root segments was extracted in 2 M HCl and Al content was determined by ICP-AES. Bars represent standard error (*n* = 3). Different lowercase letters indicate significant differences at *p* < 0.05. DW, dry weight.

Roots and RBCs accumulated substantial amounts of Al after being exposed to Al in mist. The concentration of Al in RBCs was up to 5.1 ± 0.1 mg (g dry weight)^-1^, which was 5.6-fold higher than that in 0–10.0 mm root segments (**Figures 1A,B**). The concentration of Al in 0–2.5 mm and 2.5–5.0 mm root segments was significantly higher than that in 5.0–10.0 mm root segments (**Figures 1A,B**). This demonstrated that the root apex (0–5.0 mm) is the main target for Al accumulation in mist culture. There was little difference in Al concentration between 0–2.5 mm and 2.5–5 mm root segments (**Figure 1A**); therefore, Al content was compared in 0–2.5 mm root segments and RBCs based on the number of RBCs typically found around one root apex. The number of RBCs in a set reaches up to 10^4^ per apex (Yu et al., 2006). Total Al content in one set of RBCs was approximately 24.5% of that in one root apex (**Figure 1C**), indicating that RBCs builds up an effective shield to impair Al entry into the root apex.

### Distribution of Al in Cell Wall Constituents of RBCs

**FIGURE 2:**
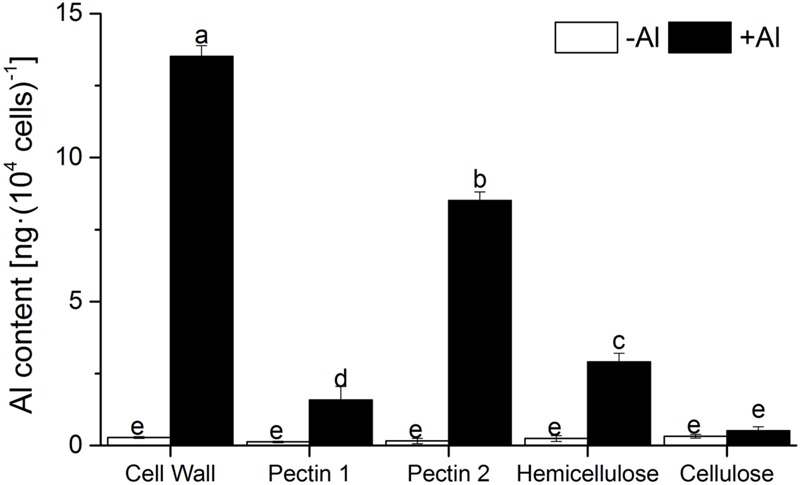
**Content of Al in sequentially extracted pellets of RBCs.** Seedlings were treated with 0 or 1.0 mM AlCl_3_ (in 0.5 mM CaCl_2_, pH 4.0) mist for 24 h. Al in pellets of RBCs was extracted in 2 M HCl and determined by ICP-AES. Bars represent standard error (*n* = 3). Different lowercase letters indicate significant differences at *p* < 0.05.

The proportion of Al in the cell wall, calculated from the Al content in whole cells (**Figure 1C**) and in the cell wall (**Figure 2**), amounted to 65.3% of that in the whole cells, implying that a minor amount of Al enters into RBCs through the cell wall. Alkali-soluble pectin, hemicellulose, and pectin 1 contained 70.4, 21, and 12% of the Al in the cell wall, respectively. Interestingly, Al binding in pectin 2 was 5.6-fold higher than that in pectin 1, indicating that pectin 2 is the primary target of Al among the cell wall constituents (**Figure 2**). There was little difference of Al content in cellulose between the -Al and +Al treatments (**Figure 2**), suggesting that there was no Al in cellulose.

### Adsorption of Al in Cell Wall Samples

**FIGURE 3:**
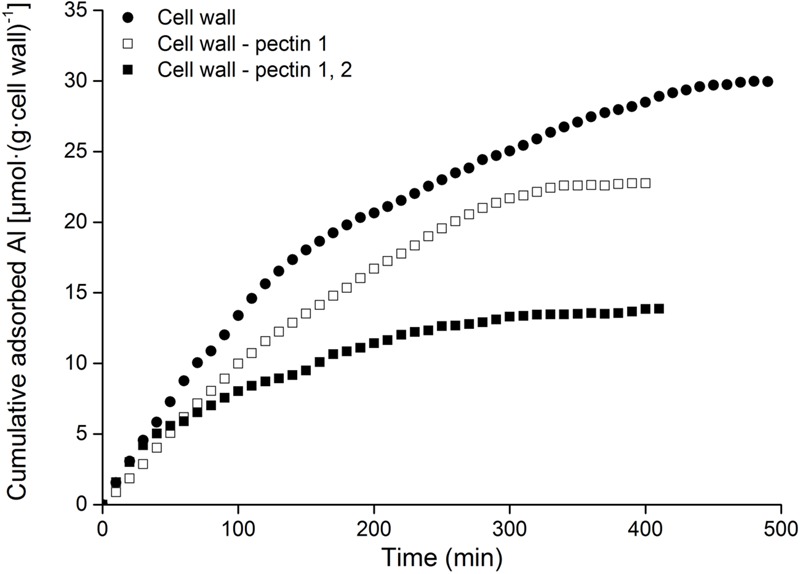
**Kinetics of Al adsorption in cell wall materials of RBCs.** The adsorption of cell wall materials was carried out using mini-column chromatography. Al content in the eluates was measured colorimetrically using pyrocatechol violet. The experiment was repeated at least three times with the same results.

Al adsorption was compared in cell wall samples following the removal of pectin 1 (Cell wall-pectin 1) and subsequently pectin 2 (Cell wall-pectin 1, 2). Al adsorption decreased with the removal of pectin 1, and it dropped sharply after the removal of pectin 2 (**Figure 3**). Al adsorption by the cell wall was reduced to 76% following the removal of pectin 1, while it was only 46.3% after further removal of pectin 2.

### Differences in Uronic Acids in Chelate-Soluble Pectin and Alkali-Soluble Pectin in RBCs

**FIGURE 4:**
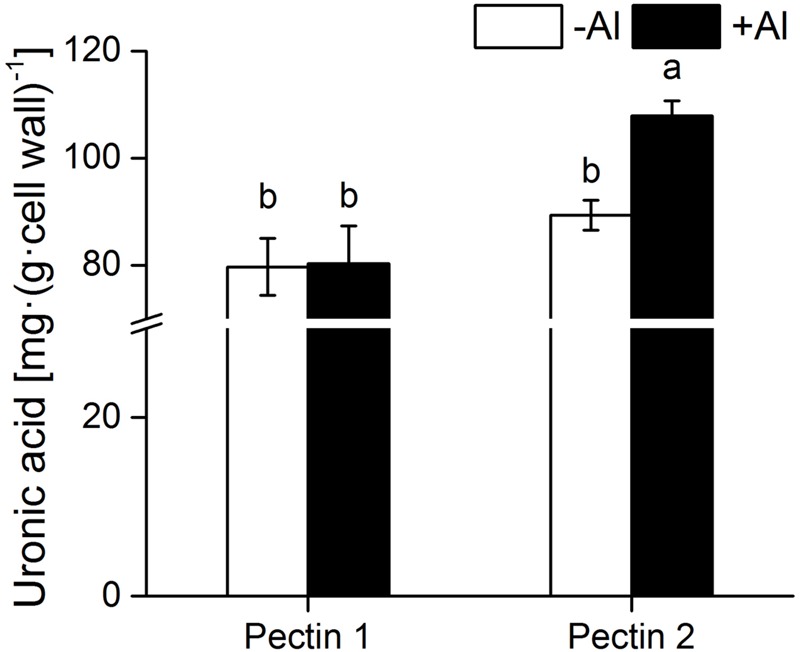
**Content of uronic acids in cell wall pectin.** Pectins 1 and 2 were extracted from cell wall samples of RBCs. The uronic acids content was determined using hydroxydiphenyl colorimetry. Different lowercase letters indicate significant differences at *p* < 0.05 for the same treatment.

Little difference was found in the content of uronic acids between pectins 1 and 2 from RBCs in the absence of Al (**Figure 4**). Al exposure significantly enhanced uronic acids content in pectin 2 but not in pectin 1. The uronic acids content in pectin 2 was about 1.5-fold greater than that in pectin 1 in the presence of Al (**Figure 4**).

### Adsorption/Desorption of Al in Pectin

Capacity of Al adsorption/desorption was compared in pectins 1 and 2 of RBCs (**Table 1**). The quantity of Al adsorption in a unit quantity of pectin 2 was 5.3-fold to that in pectin 1, indicating a significantly higher capacity for Al adsorption in pectin 2. Meanwhile, the majority of Al desorbed (68.5%) was derived from pectin 1, while a minor proportion of Al (8.5%) was desorbed from pectin 2. Thus the desorption rate (desorbed Al/adsorbed Al) of pectin 1 was 8.1-fold higher than that of pectin 2 (**Table 1**). This signifies that pectin 2 not only absorbs much more Al, but also immobilizes Al more tightly in comparison with pectin 1. In contrast, Al bound in chelate-soluble Al is more readily desorbed.

**Table 1:** Adsorption and desorption of Al in cell wall pectins 1 and 2 of RBCs.

	Al adsorption [μg⋅(g uronic acids)^-1^]	Al desorption [μg⋅(g uronic acids)^-1^]	Al desorption ratio (%)
Pectin 1	547.5 ± 21.7^b^	379.1 ± 33.1^a^	68.5 ± 5.4^a^
Pectin 2	2922.4 ± 96.6^a^	249.1 ± 28.6^b^	8.5 ± 0.7^b^

*Solutions of 30 μM AlCl_*3*_ (pH 4.5) and 0.5 mM CaCl_*2*_ (pH 4.5) were used for the adsorption and desorption processes, respectively. Al was immediately determined by a colorimetric method using pyrocatechol violet. Each value represents the mean ± standard error of at least three replicates. Different lowercase letters indicate significant differences at *p* < 0.05 within each column.*

## Discussion

### Role of Alkali-Soluble Pectin in Binding Al in the Cell Wall

Al^3+^ is so reactive that many potential cation binding sites including the cell wall, the plasma membrane surface, the cytoskeleton, and the nucleus could be the targets of Al lesion (Panda et al., 2009). We found that the majority of the Al (65.3 %) in RBCs of pea could be detected in the cell wall (**Figures 1C** and **2**). This is consistent with results from cultured tobacco cells where the ratio of Al in cell wall fractions to intact cells was 89% (Chang et al., 1999). The Al in the cell wall has two destinations. One is to continue its way to cytosol or further to vacuoles (Zhu et al., 2013), and the other is to be immobilized in the cell wall (Kopittke et al., 2009, 2015). It is generally agreed that, in comparison with the cell wall, a relative lower Al accumulation in the cytoplasm will induce greater toxicity to organelles, e.g., mitochondria, etc. (Yamamoto et al., 2002; Panda et al., 2009; Liu et al., 2014). A higher concentration of Al in the cell wall, however, may also reduce cell wall extensibility (Ma et al., 2004), inhibit cell elongation (Ma et al., 2004; Horst et al., 2010; Kopittke et al., 2015), and result in a swollen epidermis (Kopittke et al., 2015). So it is still debatable whether Al accumulation in the cell wall is an indicator of Al toxicity or tolerance/resistance (Kochian, 2003; Horst et al., 2010; Zhu et al., 2013).

Cell wall analysis showed that Al predominately accumulated in pectin, with only a small proportion in hemicellulose and no Al in cellulose (**Figure 2**). Only 46.3% of Al absorption occurred in the cell wall of RBCs after removal of pectins (**Figure 3**). It is consistent with the results in wheat (*Triticum aestivum* L.) that Al adsorption decreased by more than 50% after treatment of the cell walls with 1% pectinase for 30 min to degrade part of the pectin (Zheng et al., 2004). Therefore, cell wall pectin is the main target of Al accumulation. Both the cell wall analysis and the adsorption kinetics in the cell wall samples further disclosed that pectin 2 bound more Al than did pectin 1 (**Figures 2** and **3**). Although a lower ratio of Al was associated with pectin 1 (12%; **Figure 2**) than observed previously in cultured tobacco cells (38%; Chang et al., 1999) due to the use of a different chelator for the extraction, the majority of the Al was associated with pectin 2 in both of the RBCs of pea (70.4%, **Figure 2**) and the cultured tobacco cells (>54%, Chang et al., 1999). It is widely accepted that carboxylic groups of the pectin matrix provide the main binding sites of Al (Chang et al., 1999; Zheng et al., 2004; Yang et al., 2008, 2011; Horst et al., 2010). Therefore, two possible mechanisms, higher proportion of uronic acids (Horst et al., 2010) and higher capacity for Al adsorption by pectin (Zheng et al., 2004; Yang et al., 2008, 2011), may be involved in the increased binding of Al in pectin 2 compared with pectin 1. We found that Al toxicity induced an increase in pectin 2 but not pectin 1 in RBCs, which resulted in about 1.5-fold higher content of uronic acids in pectin 2 than in pectin 1 (**Figure 4**). It is consistent with the results in, maize (Eticha et al., 2005), rice (Yang et al., 2008), common beans (Rangel et al., 2009). However, this result could not explain why Al binding was 5.6-fold higher in pectin 2 than in pectin 1 of RBCs (**Figure 3**). The second proposed mechanism is based on the sharp discrepancy in Al adsorption and desorption in different pectins (**Table 1**). Alkali-soluble pectin had a higher capacity for Al adsorption than did pectin 1 (**Table 1**). Al adsorption in a unit of pectin 2 was 5.3-fold higher than that in a unit of pectin 1 (**Table 1**), which contributed significantly to the Al accumulation in pectin 2 of RBCs of pea. Therefore, pectin 2 represents a large pool of Al accumulation in RBCs of pea.

### Aluminum Is Immobile in Alkali-Soluble Pectin while Mobile in Chelate-Soluble Pectin

Al-induced increase in pectin, a higher content of pectin, or higher Al accumulation in cell wall pectin are usually linked to enhanced Al sensitivity (Van et al., 1994; Schols et al., 1995; Eticha et al., 2005; Horst et al., 2010; Jian Li et al., 2011; Yang et al., 2011). However, the mobility of Al in pectin might be more crucial in determining Al toxicity to cells. Our findings of Al desorption in pectins 1 and 2 may also explain the paradox of pectin in Al sensitivity and resistance. Nearly all the Al adsorbed in the cell wall could be desorbed by 2.5 mM CaCl_2_ (pH 4.5) in wheat and pea (Zheng et al., 2004; Yu et al., unpublished data), indicating that most of the Al ions were electrically bound to the cell wall materials (Zheng et al., 2004). Under the lower density of 0.5 mM CaCl_2_ (pH 4.5), we found that the majority of the Al (68.5%) adsorbed in pectin 1 was desorbed, while the desorption rate was minimal (8.5%) in pectin 2. Since Al desorbed from cell wall pectin is unlikely detoxificated by organic acids as very limited amount of its exudation was detected in root of pea whether at the absense or at the presense of Al toxicity (Yu, unpublished data). Higher Al desorption from pectin 1 in pea might be linked to higher Al sensitivity or lower Al resistance (**Table 1**; Horst et al., 2010), since it facilitates the entry of Al into the cytosol. It is inconsistet with the results in rice (Yang et al., 2011) that Al was less tightly bound in the Al-resistant cultivar than in the Al-sensitive cultivar (Yang et al., 2008), which might be associated with the finding that 75% of cell wall Al was bound in the hemicellulose instead of in pectin (Yang et al., 2011). Therefore, lower Al desorption from pectin 2 might point to the final destination of Al being immobilization in the cell wall. Interestingly, Al did not induce an increase in pectin 1 but only pectin 2 in RBCs (**Figure 4**). Therefore, pectin 2 is the primary target of Al immobilization in RBCs of pea due to its higher content of uronic acids and higher capacity of Al adsorption with lower desorption rate (**Figure 4**; **Table 1**). While pectin 1 is a small temporary store of Al in the cell wall owing to its higher desorption rate (**Figure 4**; **Table 1**). The finding of higher Al adsorption with a lower rate of desorption in pectin 2 in contrast to pectin 1 may reveal a novel mechanism of Al tolerance and sensitivity in pectins 2 and 1, respectively.

The cation (Al^3+^; Ca^2+^) binding features of pectin depend on the density of negative charges that are ultimately determined by the structure and property of pectin (Altartouri and Geitmann, 2015). Chelate-soluble pectin with low molecular mass is a readily formed product with a high level of methyl esterification, while pectin 2, having a high molecular mass, polymerizes after demethylation (Schols et al., 1995). Therefore, pectin 2 has not only more negative charges from the demethylation by pectin methyl esterase, but also a higher charge density than pectin 1. The “egg-box” structure formed in pectin with low methyl esterification (pectin 2; Braccini and Pérez, 2001) will also guarantee a higher charge density. Once Ca^2+^ (here Al^3+^) falls into the “dimple” of the pectin “egg-box” (Braccini and Pérez, 2001), it is tightly bound and forms structural pectin to maintain the stability of the cell wall, thus Al bound in pectin 2 has less mobility than that in pectin 1.

### RBCs Protect Root Apices from Al Toxicity by Immobilizing Al in Alkali-Soluble Pectin

Much evidence has been provided that RBCs could protect root tips from Al toxicity (Miyasaka and Hawes, 2001; Xing et al., 2008; Yu et al., 2009). Root growth was significantly inhibited by exposure to AlCl_3_ only when border cells were removed, while in the presence of RBCs, Al did not affect root growth (Yu et al., 2009). At the same time, RBCs were shown to maintain a high viability at millimole levels of Al, while micromole levels of Al could induce inhibition of root elongation (Kochian, 2003; Yu et al., 2009). Our present research provided further evidence for the protective roles of RBCs. In this study, the Al content in RBCs was 5.6-fold higher than that in root segments, indicating the higher capacity of Al binding in RBCs (**Figure 1**). A set of RBCs (10^4^ cells) accumulated approximately 24.5% of the total Al in one root tip (**Figure 1C**). Although 24.5% Al accumulation does not seem high for one set of RBCs, a new set of RBCs would be produced within 24 h of the old ones dying, being sloughed off from the root tips by mechanical abrasion during root elongation, or being dispersed in water/soil solution (Hawes et al., 2000). Therefore, we can conclude the significant role of RBCs in protecting root tips from Al toxicity. It could be inferred that RBCs would strive to incur as little damage as possible since they form a layer of single cells around root tips. The question is how do RBCs coordinate the protection of root apices and self-defense? Under Al toxicity, the strategy of RBCs to provide a shield to root tips as well as to their own cytosol relies on enhancing the content of pectin 2, which provides more binding sites with strong immobilization, and maintaining a relatively constant lower content of pectin 1.

### Possible Al Binding Sites of Other Cell Wall Constituents

Our results showed that hemicellulose could accumulate certain amounts of Al in RBCs. This supported findings in rice and *Arabidopsis* that considerable Al was bound in hemicellulose (Jian Li et al., 2011; He et al., 2015; Ma et al., 2015). However, the amount of Al that accumulated in hemicellulose of RBCs of pea was relatively small, contrasting with *Arabidopsis* in which hemicellulose was the major pool for Al accumulation (based on the fact that about 75% of cell wall Al accumulated in the hemicellulose). This shows that hemicellulose may accumulate Al to different extents depending on plant species and cultivar.

## Conclusion

Al targets multiple cellular sites simultaneously, and pectin 2 is the primary pool of Al immobilization, which contributes to Al tolerance of plant cells, while pectin 1 acts as a small, temporary Al store that confers Al sensitivity in plant cells. RBCs have developed a strategy to fix more Al in pectin 2 and bind less Al in pectin 1, protecting themselves and preventing the entrance of toxic Al ions into the young root cells.

## Author Contributions

JY and MQ performed the analysis of Al in cell wall components and wrote the manuscript. JF carried out the adsorption/desorption of Al in the cell wall and pectin. RS and LW detected Al in the samples with ICP-AES. YF and JL cultured RBCs and measured the content of uronic acids. JB and YH detected Al content in roots and RBCs. MY designed the research and revised the manuscript.

## Conflict of Interest Statement

The authors declare that the research was conducted in the absence of any commercial or financial relationships that could be construed as a potential conflict of interest.

## Abbreviations

ICP-AES: inductively coupled plasma atomic emission spectrometry
Pectin 1: chelate-soluble pectin
Pectin 2: alkali-soluble pectin
RBCs: root border cells
